# Supplementary material for: ATP biphasically modulates LLPS of TDP-43 PLD by specifically binding arginine residues
Source: Commun Biol. 2021 Jun 10;4:714. doi: 10.1038/s42003-021-02247-2 (PMC8192790; doi:10.1038/s42003-021-02247-2)
Supplement: Supplementary file 2 — Supplementary Information [file 42003_2021_2247_MOESM2_ESM.pdf]

ATP biphasically modulates LLPS of TDP-43 PLD by specifically binding arginine residues

Dang Mei[1], Liangzhong Lim[1], Jian Kang[1] and Jianxing Song[1]*
[1]Department of Biological Sciences, Faculty of Science; National University of Singapore; 10 Kent Ridge Crescent, Singapore 119260

Supplementary Figures 1-5

Supplementary Figure 1. Unique hydration structure of ATP.

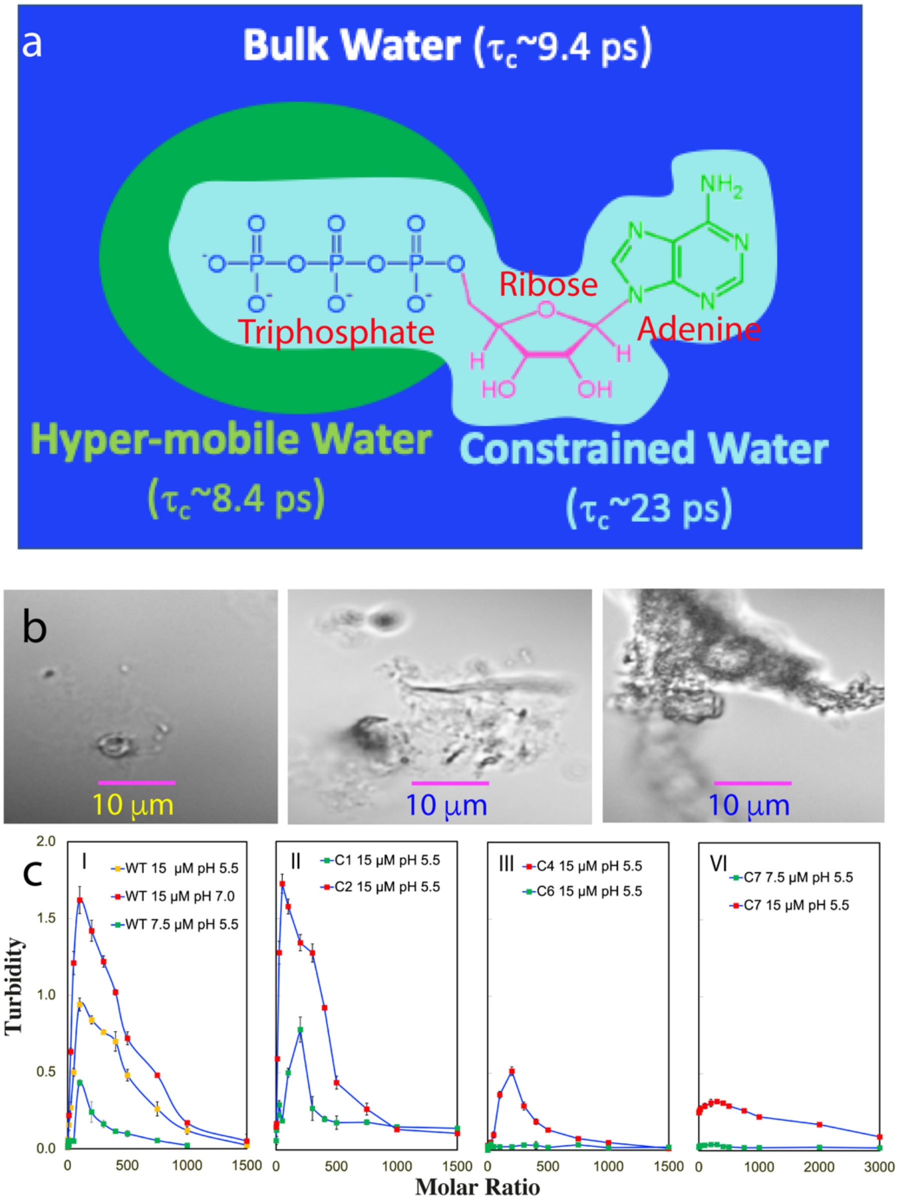

(a) The unique hydration structure of ATP previously revealed by microwave dielectric spectroscopy (Ref. 24), which contains the "constrained water" with dielectric relaxation time (τc) of ~23 ps, as well as the "hyper-mobile water" with τc of ~8.4 ps, smaller than that of bulk water (9.4 ps). (b) DIC images of aggregated and precipitated samples. (c) (I) Turbidity of TDP-43 PLD at 15 μM in the presence of ATP at different molar ratios in 10 mM sodium phosphate buffer at pH 5.5 and 7.0 as well as at 7.5 μM and pH 5.5. (II) Turbidity curves of TDP-43 C1- and C2-PLD at 15 μM in the presence of ATP at different molar ratios in 10 mM sodium phosphate buffer at pH 5.5. (III) Turbidity curves of TDP-43 C4- and C6-PLD at 15 μM in the presence of ATP at different molar ratios in 10 mM sodium phosphate buffer at pH 5.5. (VI) Turbidity curves of TDP-43 C7-PLD at 15 μM and 7.5 μM in the presence of ATP at different molar ratios in 10 mM sodium phosphate buffer at pH 5.5.

Supplementary Figure 2. Characterization of the interaction of TDP-43 PLD with AMP.

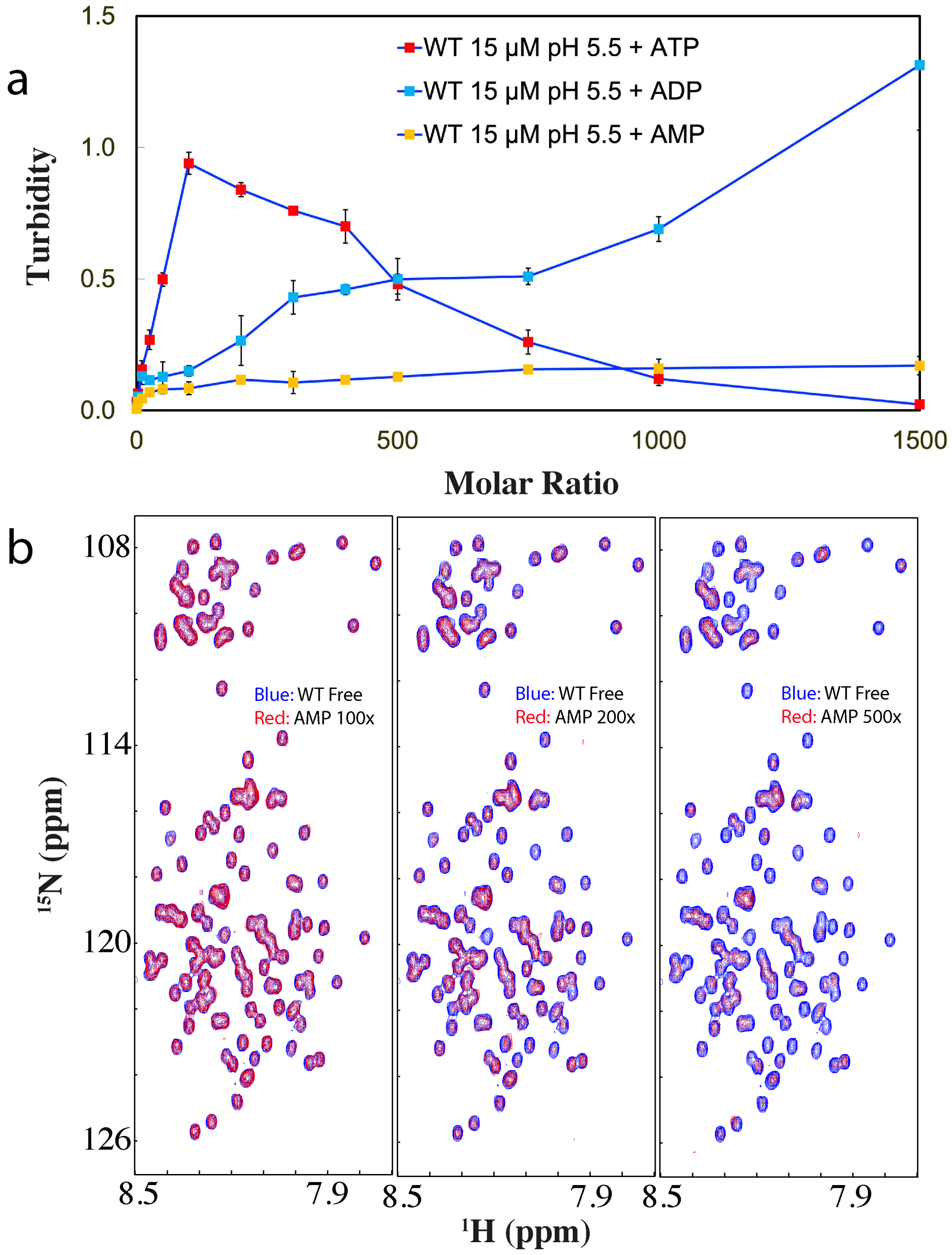

(a) Turbidity curves of TDP-43 PLD at 15 μM in the presence of ATP, ADP and AMP at 1;2.5, 1:10, 1:25, 1:50, 1:100, 1:200, 1:300, 1:400, 1:500, 1:750, 1:100 and 1:1500 in 10 mM sodium phosphate buffer (pH 5.5). (b) HSQC spectra of the ^{15}N-labeled TDP-43 PLD at 15 μM in the absence (blue) and in the presence of AMP (red) at different molar ratios in the same buffer at pH 5.5.

Supplementary Figure 3. Characterization of the interaction of TDP-43 PLD with ADP.

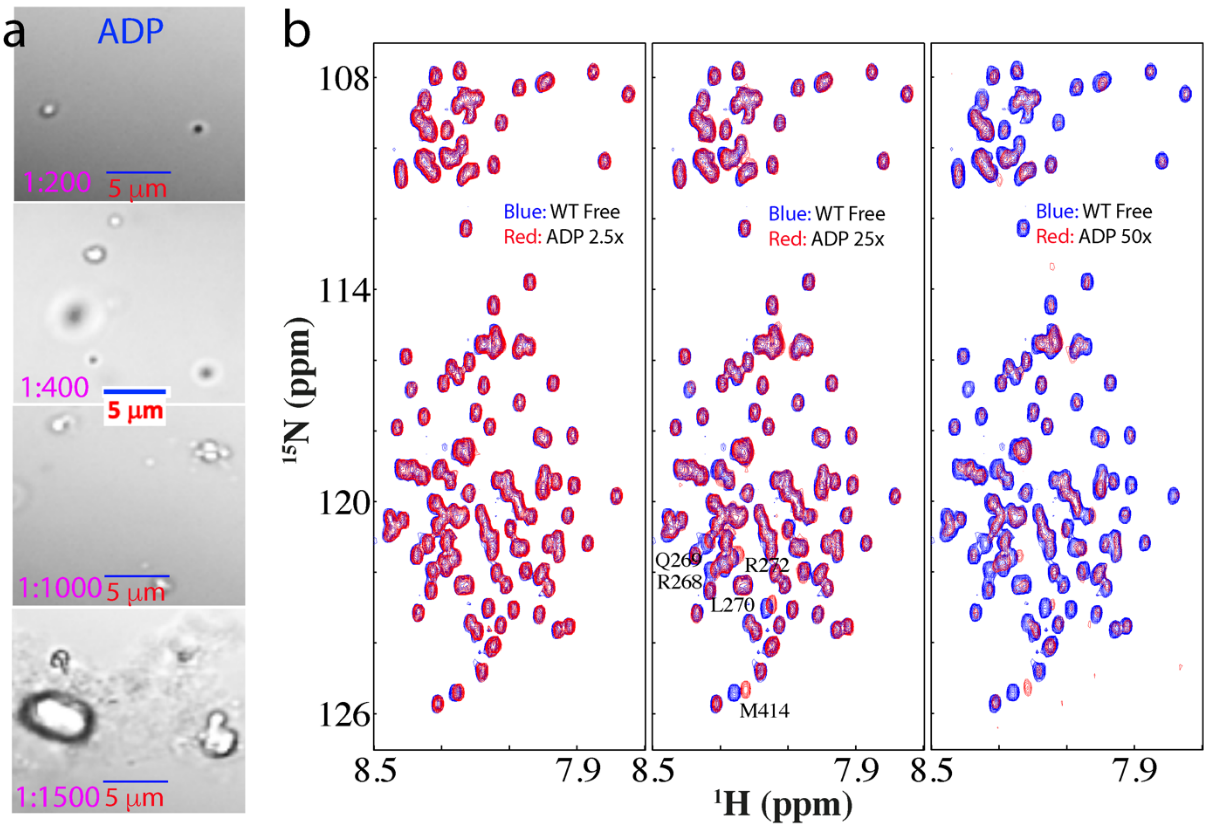

(a) DIC images of TDP-43 PLD at 15 μM in the presence of ADP at different molar ratios in 10 mM sodium phosphate buffer (pH 5.5). (b) HSQC spectra of the ^{15}N-labeled TDP-43 PLD at 15 μM in the absence (blue) and in the presence of ADP (red) at different molar ratios in the same buffer at pH 5.5.

Supplementary Figure 4. NMR characterization of TDP-43 PLD mutants C1 to C3.

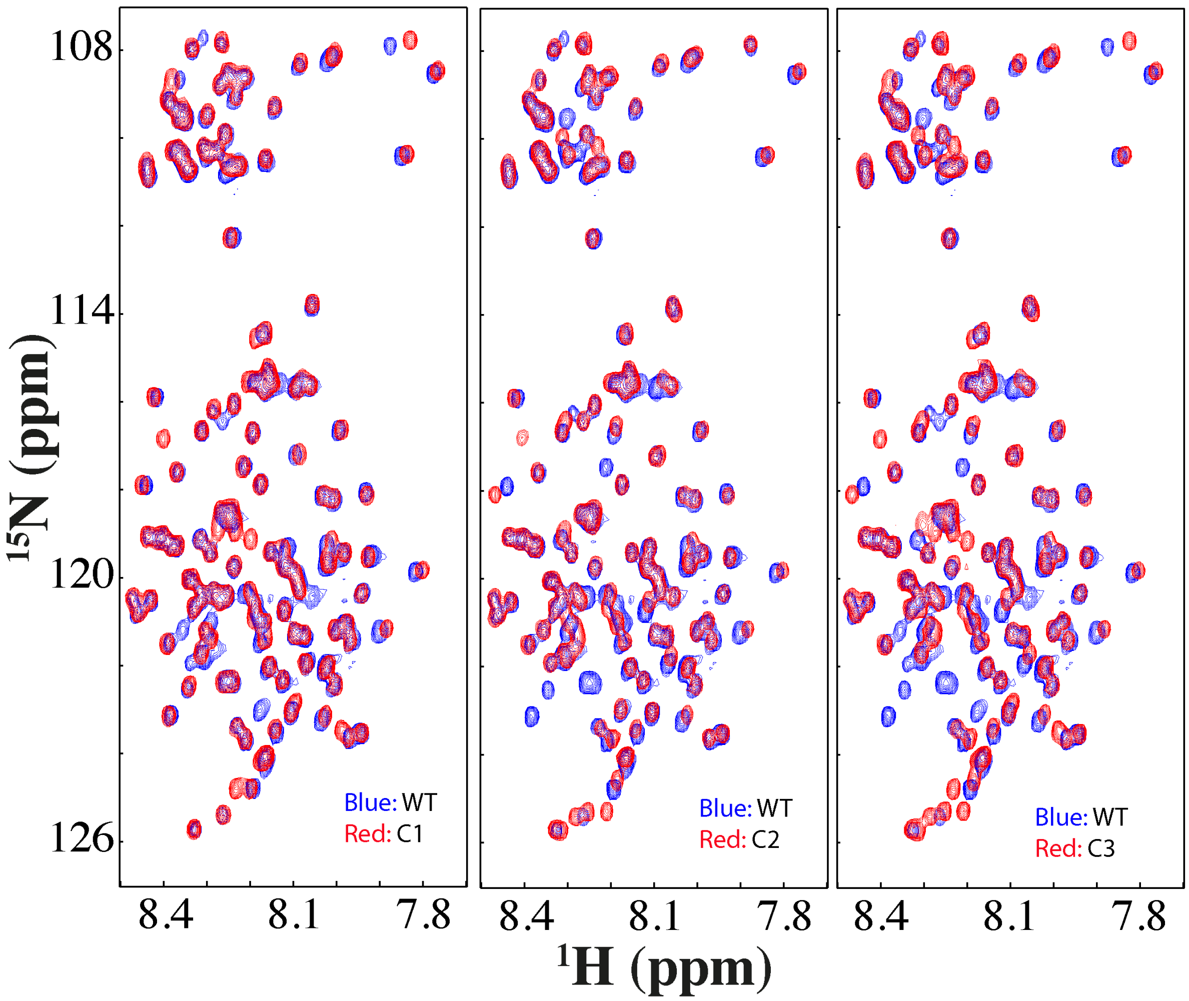

HSQC spectra of the ^{15}N-labeled TDP-43 WT-PLD (blue) and C1-, C2- and C3-PLD (red) at 15 µM in 10 mM sodium phosphate buffer (pH 5.5).

Supplementary Figure 5. NMR characterization of TDP-43 PLD mutants C4-C7.

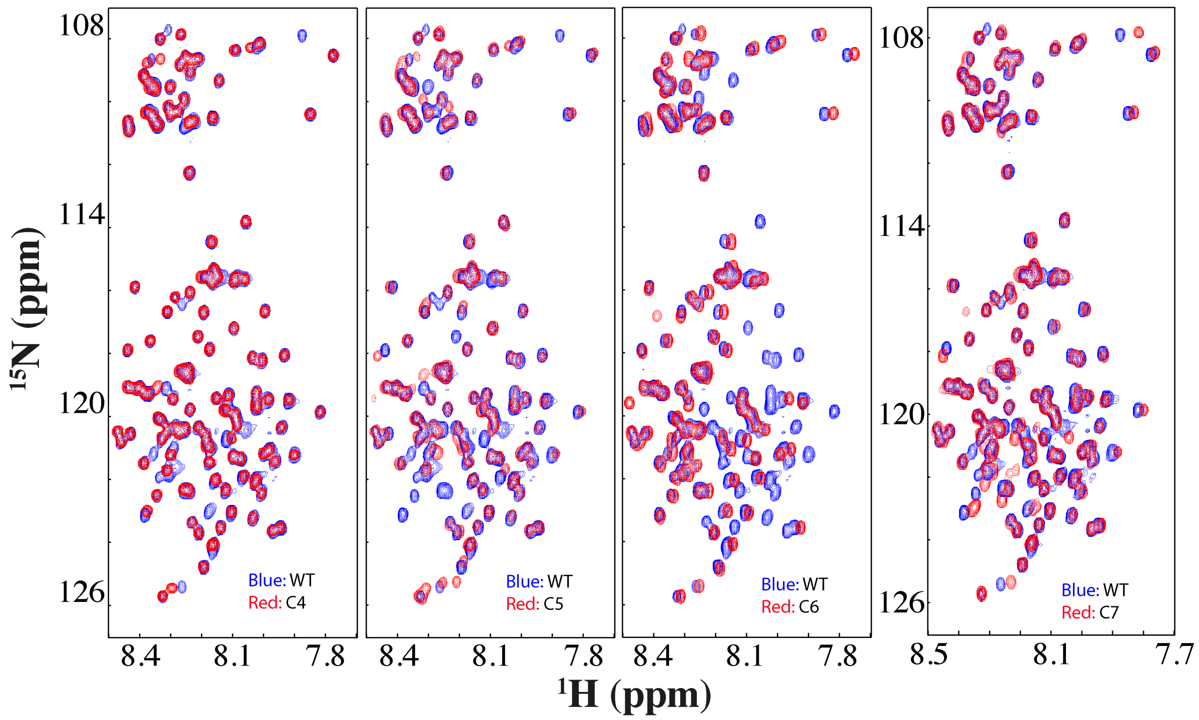

HSQC spectra of the ^{15}N-labeled TDP-43 WT-PLD (blue) and C4-, C5-, C6- and C7-PLD (red) at 15 μM in 10 mM sodium phosphate buffer (pH 5.5).
