# Supplementary material for: ATP biphasically modulates LLPS of TDP-43 PLD by specifically binding arginine residues
Source: Commun Biol. 2021 Jun 10;4:714. doi: 10.1038/s42003-021-02247-2 (PMC8192790; doi:10.1038/s42003-021-02247-2)
Supplement: Supplementary file 3 — Description of Supplementary Files [file 42003_2021_2247_MOESM3_ESM.pdf]

Description of Additional Supplementary Files

File name: Supplementary Data 1

Description: Source data for all graphs and charts.
